# Supplementary material for: Uncovering the Molecular Interactions Underlying MBD2 and MBD3 Phase Separation
Source: J Phys Chem B. 2025 May 12;129(23):5728–43. doi: 10.1021/acs.jpcb.5c02741 (PMC12169658; doi:10.1021/acs.jpcb.5c02741)
Supplement: Supplementary file 1 [file jp5c02741_si_001.pdf]

SUPPORTING INFORMATION

Uncovering the Molecular Interactions Underlying MBD2 and MBD3 Phase Separation

Nicole Maurici[1*], Tien M. Phan[2*], Jessica L. Henty-Ridilla[1,3], Young C. Kim[4], Jeetain Mittal[2,5,6#], Alaji Bah[1#]

[1]*Department of Biochemistry and Molecular Biology, SUNY Upstate Medical University, Syracuse, NY, 13210*
[2]*Artie McFerrin Department of Chemical Engineering, Texas A&M University, College Station, TX, 77843*
[3]*Department of Neuroscience and Physiology, SUNY Upstate Medical University, Syracuse, NY, 13210*
[4]*Center for Materials Physics and Technology, Naval Research Laboratory, Washington, DC, 20375*
[5]*Department of Chemistry, Texas A&M University, College Station, TX, 77843*
[6]*Interdisciplinary Graduate Program in Genetics and Genomics, Texas A&M University, College Station, TX, 77843*

*Co-first authors
[#]To whom correspondence may be addressed: jeetain@tamu.edu, baha@upstate.edu

METHODS

Step-by-step protocol for the expression and purification of full-length MBD2 and MBD3 and their truncation variants

This protocol outlines the expression and purification of full-length MBD2 and MBD3. As the procedure is identical for both proteins, we describe the process for MBD2 only to minimize redundancy. This method yields milligram quantities of highly purified protein from 1 L of bacterial culture.

Note: All cysteine residues in MBD2 and MBD3 were mutated to serine via site-directed mutagenesis to prevent disulfide bond formation and reduce aggregation. Control experiments confirmed that these cysteine-less variants retain the DNA-binding activity and phase separation behavior of the wild-type proteins.

Cloning and Transformation

1. Clone codon-optimized cDNA of full-length MBD2 (with cysteine-to-serine mutations) into a pET28 vector with kanamycin resistance, containing an N-terminal His[illegible]-SUMO tag. The SUMO tag enhances solubility and enables cleavage with no residual amino acid using the SUMO-specific protease Ulp1.
2. Transform the plasmid into chemically competent *E. coli* BL21-CodonPlus (DE3) RIPL cells.
3. Plate transformed cells on LB agar containing 50 µg/mL kanamycin and 34 µg/mL chloramphenicol. Incubate overnight at 37 °C.

Starter Culture and Expression

4. Inoculate a 25 mL LB starter culture (with appropriate antibiotics) using a single colony. Incubate overnight at 37 °C with shaking.
5. Inoculate 1 L of LB media (in a 2.5 L baffled Erlenmeyer flask) with 12.5 mL of the overnight culture. Grow at 37 °C, 220–250 rpm until OD[illegible] reaches ~1.0 (approximately 4 hours).
6. Induce protein expression by adding IPTG to a final concentration of 1 mM. Shift the temperature to 16 °C and incubate overnight (12–16 hours).

Cell Harvesting and Lysis

7. Pellet cells by centrifugation at 4000 rpm for 30 min at 4 °C. Discard the supernatant.
8. Resuspend the pellet in 50 mL lysis buffer (300 mM NaCl, 50 mM Na[illegible]PO[illegible] pH 7.4, 4 M guanidinium hydrochloride, 5 mM imidazole). Store at –80 °C or proceed to lysis.
9. Thaw the cell suspension on ice and bring the total volume to 200 mL with lysis buffer. Homogenize by stirring or vortexing as needed.
10. Sonicate the lysate on ice (e.g., 2 seconds on, 4 seconds off for 15 minutes) to ensure thorough lysis.

Affinity Purification and Elution

11. Clarify the lysate by centrifugation at 15,000 rpm for 30 minutes at 4 °C.
12. Incubate the supernatant with 25 mL Ni-NTA resin for 15–20 minutes at 4 °C with gentle rocking.
13. Load the mixture onto a gravity column. Collect the flowthrough.
14. Wash the column with 2 column volumes (CV) of lysis buffer, followed by 3 CV of wash buffer (300 mM NaCl, 50 mM Na□PO□ pH 7.4, 200 mM arginine, 10% glycerol, 5 mM imidazole).
15. Elute bound protein using five 10-mL fractions of elution buffer (same as wash buffer but with 500 mM imidazole).
16. Analyze all fractions (flowthrough, washes, elutions) via 15% SDS-PAGE. Pool the purest elution fractions.

SUMO Cleavage and Final Purification

17. Dialyze the pooled protein overnight at 4 °C against 4 L of buffer (300 mM NaCl, 50 mM Na□PO□ pH 7.4, 200 mM arginine, 10% glycerol, 5 mM imidazole), while simultaneously adding Ulp1 protease to cleave the SUMO tag.
18. Confirm tag removal via SDS-PAGE by comparing pre- and post-cleavage samples.
19. Concentrate the cleaved protein to ~10 mL using an appropriate molecular weight cut-off concentrator.
20. Load the concentrated protein onto a HiLoad 26/60 Superdex 75 column equilibrated in the same buffer.
21. Collect and analyze fractions by SDS-PAGE. Pool fractions containing highly pure, tag-free protein.
22. Aliquot the purified protein and store at –80 °C.

SUPPORTING INFORMATION FIGURES

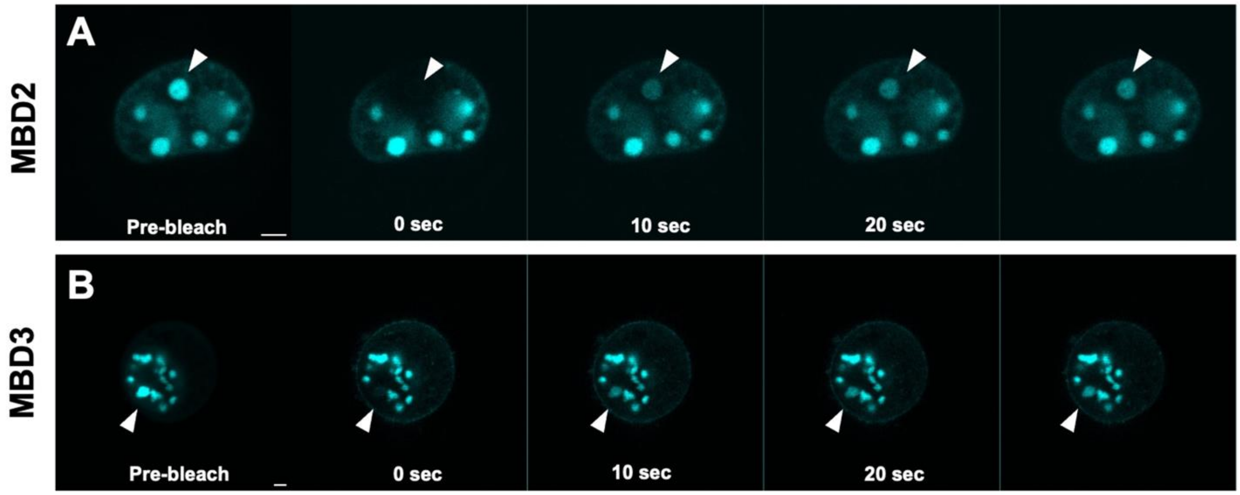

Figure S1. Pre- and post-fluorescence recovery after photobleaching (FRAP) images of live NIH-3T3 cells transfected with **A**. eGFP-tagged full-length MBD2 **B**. eGFP-tagged full-length MBD3. MBD2 and MBD3 droplet recovery is monitored from left to right as indicated by a white arrow within a 30-second time frame. Protein droplets were bleached using a 488 nm laser at 50% laser power. All cells are viewed at 100X using a Leica SP8 confocal microscope. The scale bar, represented by a white bar on the bottom right corner of the pre-bleach images, is 2 microns. Image montages were generated using FIJI/ImageJ.

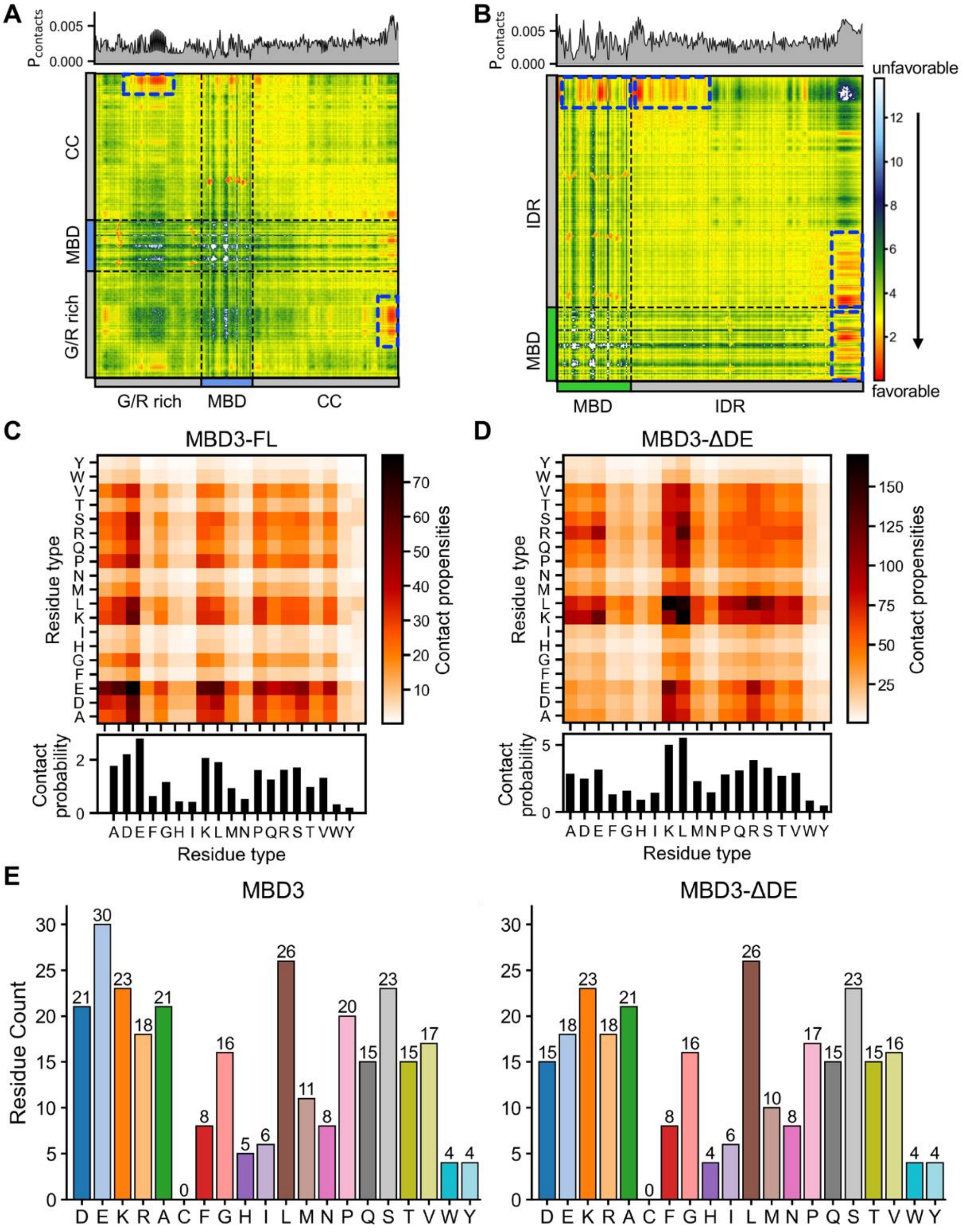

Figure S2. **A and B.** Intermolecular contacts of MBD2 and MBD3 proteins within the condensed phase. Preferential interactions are shown in red. The 1D contact map on the top is the average contact propensity per frame per residue. The CG coexistence simulations were conducted using the HPS-Urry model at 320 K and 100 mM salt concentration. Blue boxes highlight dominant contact-prone regions. **C and D**. Intermolecular contact maps within the condensate between two residue types within the condensate formed by FL MBD3 and its ΔDE truncation, respectively. **E**. Bar charts of amino acid abundance in FL MBD3 and its ΔDE truncation.

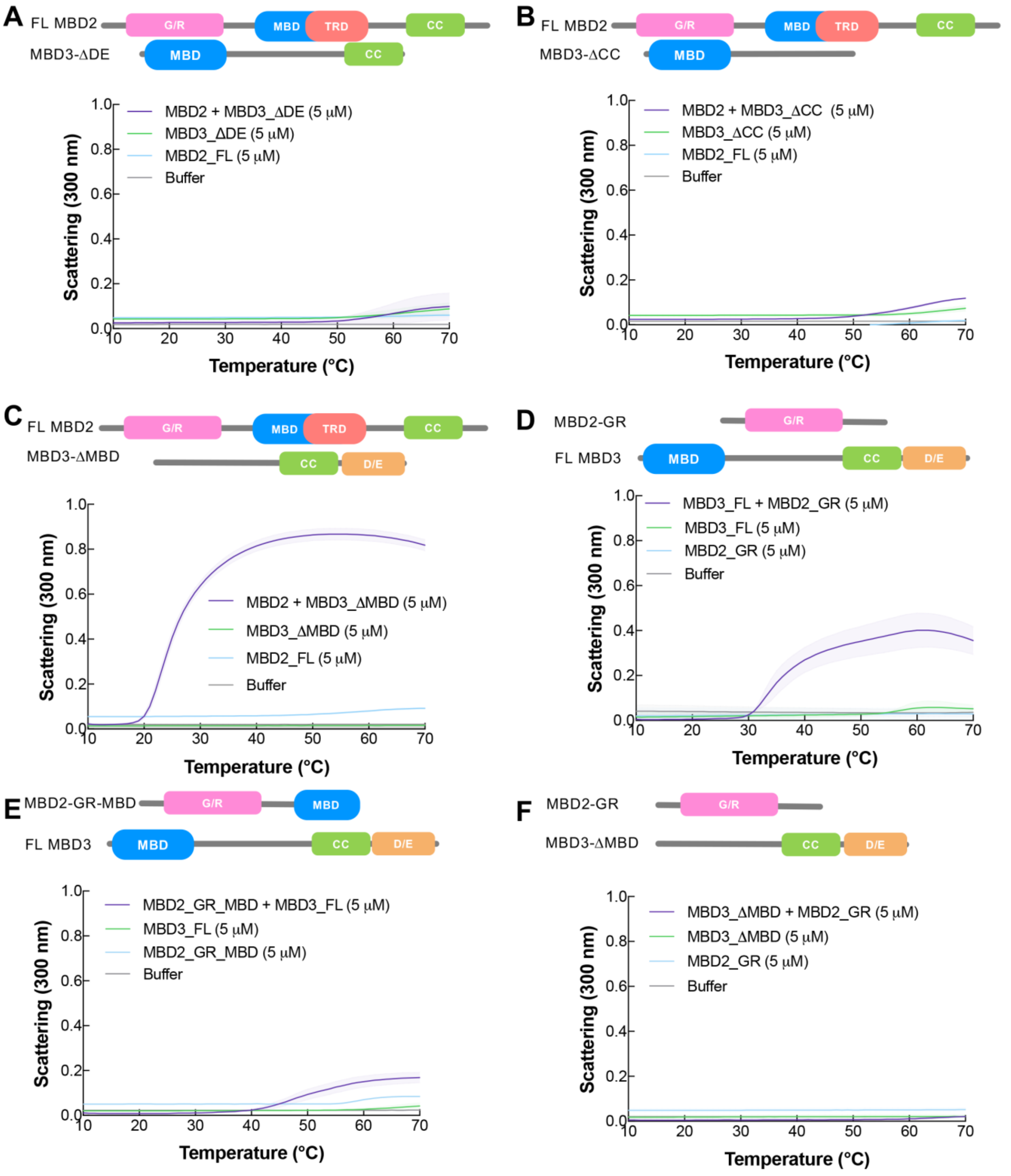

Figure S3. UV-Vis absorption spectra of **A**. FL MBD2 and ΔDE **B**. FL MBD2 and ΔCC **C**. FL MBD2 and ΔMBD **D**. FL MBD3 and GR **E**. FL MBD3 and GR-MBD **F**. GR and ΔMBD. The spectra show absorption as a function of the temperature of each protein individually (5 μM) and mixed at an equimolar ratio (final concentration of 5 μM) in a phase separation buffer. The shading around each curve represents the standard deviation from the mean absorbance from technical replicates.

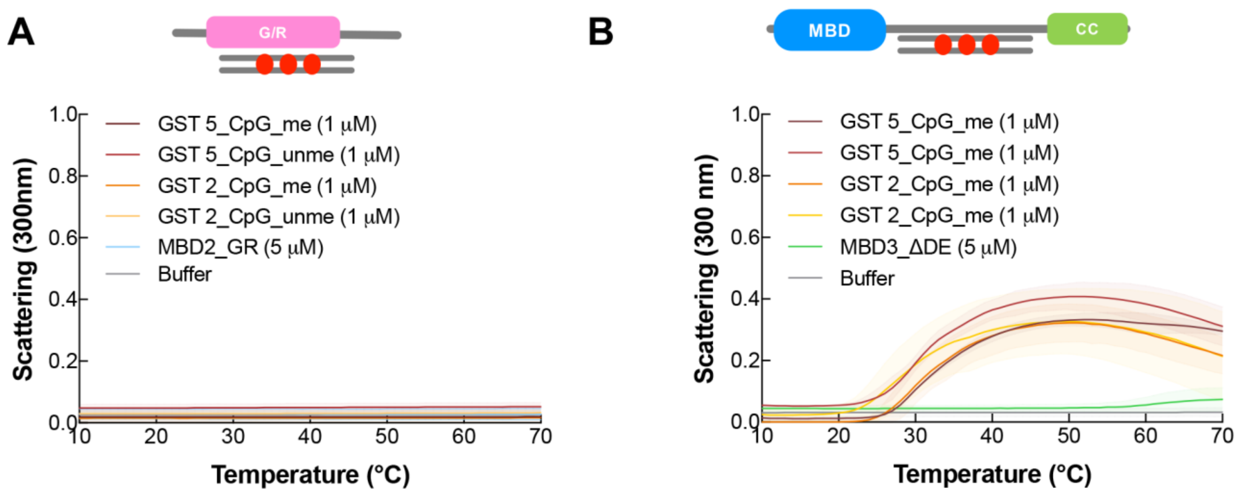

Figure S4. UV-Vis absorption spectra of **A**. GR and **B**. ΔDE individually and mixed with either unmethylated or methylated DNA that contains either 2 or 5 CpG sites in phase separation buffer as a function of temperature. Protein and DNA concentrations remained constant at 5 and 1 μM, respectively. The shading around each curve represents the standard deviation from the mean absorbance from technical replicates.

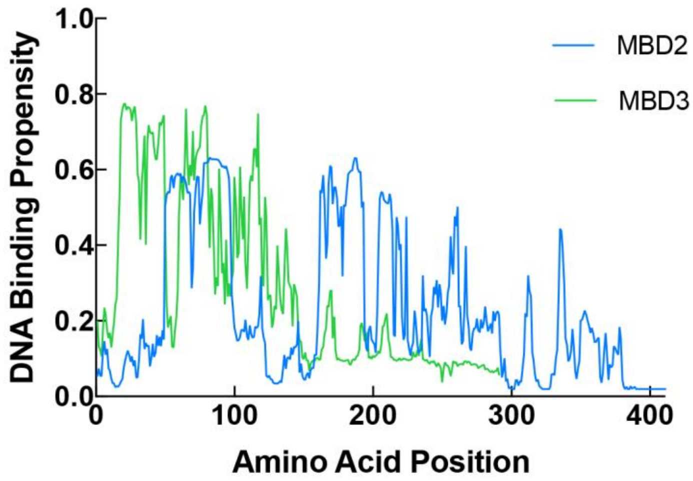

Figure S5. HybridDBRpred DNA binding propensity predictions for MBD2 and MBD3. A DNA binding propensity score above 0.28 indicates binding to DNA.
